# A slow-cooling-rate *in situ* cell for long-duration studies of mineral precipitation in cold aqueous environments on Earth and other planetary bodies

**DOI:** 10.1107/S1600576718008816

**Published:** 2018-07-26

**Authors:** Stephen P. Thompson, Hilary Kennedy, Sarah J. Day, Annabelle R. Baker, Benjamin M. Butler, Emmal Safi, Jon Kelly, Andrew Male, Jonathan Potter, Tom Cobb, Claire A. Murray, Chiu C. Tang, Aneurin Evans, Ronaldo Mercado

**Affiliations:** a Diamond Light Source, Harwell Science and Innovation Campus, Didcot, Oxfordshire OX11 0DE, UK; b School of Ocean Sciences, Bangor University, Menai Bridge, Anglesey LL59 5AB, UK; c Environmental and Biochemical Sciences, The James Hutton Institute, Craigiebuckler, Aberdeen AB15 8QH, UK; d Astrophysics Group, Lennard-Jones Laboratories, Keele University, Keele, Staffordshire ST5 5BG, UK

**Keywords:** long-duration studies, mineral precipitation, cold aqueous environments, terrestrial minerals, planetary minerals, planetary ices, solar system

## Abstract

Liquid oceans and ice caps, along with ice crusts, have long been considered defining features of the Earth, but space missions and observations have shown that they are in fact common features among many of the solar system’s outer planets and their satellites. Interactions with rock-forming materials have produced saline oceans not dissimilar in many respects to those on Earth, where mineral precipitation within frozen seawater plays a significant role in both determining global properties and regulating the environment in which a complex ecosystem of extremophiles exists. Since water is considered an essential ingredient for life, the presence of oceans and ice on other solar system bodies is of great astrobiological interest. However, the details surrounding mineral precipitation in freezing environments are still poorly constrained, owing to the difficulties of sampling and *ex situ* preservation for laboratory analysis, meaning that predictive models have limited empirical underpinnings. To address this, the design and performance characterization of a transmission-geometry sample cell for use in long-duration synchrotron X-ray powder diffraction studies of *in situ* mineral precipitation from aqueous ice–brine systems are presented. The cell is capable of very slow cooling rates (*e.g.* 0.3°C per day or less), and its performance is demonstrated with the results from a year-long study of the precipitation of the hydrated magnesium sulfate phase meridianiite (MgSO_4_·11H_2_O) from the MgSO_4_–H_2_O system. Evidence from the Mars Rover mission suggests that this hydrated phase is widespread on the present-day surface of Mars. However, as well as the predicted hexagonal ice and meridianiite phases, an additional hydrated sulfate phase and a disordered phase are observed.

## Abstract 2

A sample environment cell for long-duration X-ray diffraction studies of mineral precipitation in very slow cooling aqueous environments on Earth and other planetary bodies is described. The results are reported of a year-long commissioning experiment monitoring the dynamics of a freezing MgSO_4_–H_2_O solution at −28°C (245 K) in which meridianiite and epsomite are observed to form.

## 1. Introduction   

### 1.1. Low-temperature aquatic mineral precipitation   

Cold aqueous environments play a critical role in defining surface geochemistry, both on Earth and on certain other planetary bodies in our solar system (*e.g.* Mars, and the icy satellites of Jupiter and Saturn; see Table 1 ▸) where water is found in close association with ice (Marion & Farren, 1999 ▸; Hussmann *et al.*, 2006 ▸; Massé *et al.*, 2014 ▸; Nimmo & Pappalardo, 2016 ▸). For example, the freezing of aqueous solutions containing dissolved salts such as NaCl, KCl, Na_2_SO_4_, MgCl_2_ and CaCl_2_ – the main dissolved salts in terrestrial oceans – results in the formation of highly concentrated electrolyte solutions (brines) caused by the separation of pure water into solid ice *via* eutectic freezing (Cox & Weeks, 1983 ▸). The precipitation of minerals within such ice–brine environments means that they tend to be retained rather than lost to the underlying water column (Light *et al.*, 2003 ▸) and are therefore retained close to the ice–atmosphere (or ice–space) interface. The presence of mineral precipitates affects the structural and optical properties of the ice as a result of their size distribution and their regulatory effect on brine volume (Assur, 1960 ▸; Maykut & Light, 1995 ▸; Light *et al.*, 2003 ▸, 2009 ▸), which in turn may influence global properties such as albedo and the effect that varying insolation has on climate change (*e.g.* Cheng *et al.*, 2009 ▸).

Understanding the chemical and physical properties of mineral precipitation from highly concentrated electrolyte solutions is thus fundamental to understanding the precise role played by planetary aquatic processes: in particular, their contribution to climate change on Earth (both present and historical, since the planet cycles between greenhouse, icehouse and possibly snowball phases), present and past hydrological activity on Mars, and core–ocean–ice crust interactions on bodies such as Europa and Enceladus where saline subsurface oceans exist beneath a surface ice crust (*e.g.* Hussmann *et al.*, 2006 ▸). These latter ocean worlds are also considered to be highly plausible habitats for extraterrestrial life and, after the successful Cassini mission to Saturn, are the targets of future planned space missions (*e.g.* NASA Ocean Worlds program, ESA JUICE mission). Indeed, on Earth, sea ice brine channels host a wide range of sympagic organisms (bacteria, microalgae, viruses, fungi, protozoans and metazoans) where osmotic conditions (salinity) are defined by temperature-dependent salt precipitation in the Na–K–Mg–Ca–Cl–SO_4_–H_2_O sea water system [*e.g.* the precipitation of mirabilite Na_2_SO_4_·10H_2_O below −6.4°C (266.8 K); Butler *et al.*, 2016 ▸]. Mineral precipitates can also provide solid surfaces for biogenic interactions (Cleaves *et al.*, 2012 ▸), and in the context of the precursor-RNA-world hypothesis for the abiogenic origin of life, cold ice–brine environments may have been an essential low-temperature step in the early replication of nucleic acids (Trinks *et al.*, 2005 ▸; Price, 2007 ▸; Vincent *et al.*, 2004 ▸; Feller, 2017 ▸). It would seem plausible to assume that cryogenic brines and their low-temperature precipitates could play (or could have played) a similar role for low-temperature biogenic reactions in the development of putative life in the low-temperature environments extant, for example, on Mars, Europa or Enceladus (Price, 2007 ▸, 2010 ▸).

### 1.2. Limitations of current models and *ex situ* measurements   

Mathematical models, such as *FREZCHEM* (Marion & Kargel, 2008 ▸), use the basic physical–chemical thermodynamic properties of multicomponent solutions to predict the chemical composition of aqueous solutions at temperatures down to −113°C (160 K) and pressures up to 0.1 MPa, suitable for investigation of environmental settings on Earth, Mars and the icy satellites (Marion & Farren, 1999 ▸; Marion *et al.*, 2003 ▸; Kargel *et al.*, 2000 ▸). Although theoretical models have been used to predict the changing chemistry of sea ice brines on Earth and other planets during freezing, there is very little *in situ* field data to support the predictions. A further limitation to modelling is that predictions of the chemical composition of electrolyte solutions at sub-zero temperatures are rarely made at sub-zero temperatures. Our understanding of chemical equilibrium in cryogenic brines can thus be limited by large uncertainties, due to the estimation through extrapolation of the existing salinity–temperature functions down to the physical conditions for temperatures below 0°C (273 K) and salinities above 50 g kg^−1^ (Papadimitriou *et al.*, 2016 ▸). In addition, thermodynamic modelling may not predict variations in solution chemistry, or mineralogy, in natural settings at sub-zero temperatures, since metastable states can both exist and, in some cases, persist, while environmental changes can often occur rapidly over timescales that are shorter than those required to reach thermodynamic equilibrium (Ewert & Deming, 2013 ▸).

Even on Earth there are problems with obtaining reliable field data. The inhospitable conditions that occur during the austral winter limit the *in situ* sampling and analysis of sea ice. Although retrieval of cores may be possible, post-collection changes in storage temperature and the methodology used to separate minerals from ice may result in changes, such that the sample under investigation no longer represents the environment from which it was extracted. Exploration of other planetary bodies necessitates the use of landers, with limited sampling and analysis capabilities, or spectroscopic remote sensing *via* spacecraft flyby missions, which have limited spatial resolution and surface penetration.

Laboratory simulations, on the other hand, offer the prospect of investigating low-temperature phases in a controlled environment. However, compromises are often made because of managerial constraints, such as time-limited experimental manipulations, access to facilities or operational constraints such as the minimum cooling rates achievable by the experimental apparatus. At worst, this can mean experimental outcomes are driven by kinetics rather than thermodynamics, such that the end-point phases obtained are not those that would arise from thermodynamically driven systems, or that crucial intermediary phases are not observed (Butler *et al.*, 2017 ▸).

### 1.3. Investigating aqueous precipitation at the Diamond Long-Duration Experiments facility   

**Figure 1:**
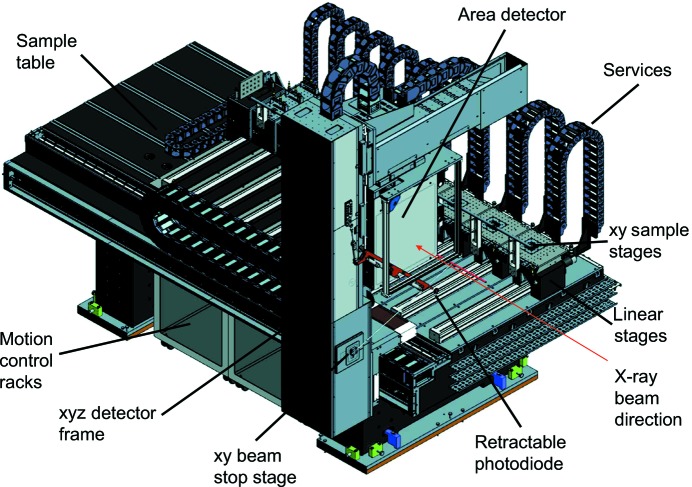
CAD drawing of the I11 Long-Duration Experiments sample table, showing the main components. Each sample stage hosts an individual experiment, and the area detector moves along the table to a pre-defined distance as each experiment is brought successively in and out of the X-ray beam (see Murray *et al.*, 2017 ▸, for full details).

The recently constructed Long-Duration Experiments Facility (LDE) on Beamline I11 at the Diamond Light Source has been designed specifically to address the issue of long-term access to structure-probing facilities for slowly evolving systems, and is an ideal platform on which to conduct experiments and measurements of mineral precipitation in cold aquatic environments. The I11 LDE has been described in detail by Murray *et al.* (2017 ▸); in brief, the facility houses multiple long-running experiments (*e.g.* 6 months to 2 years), each mounted on individual motorized *xy* stages, which are mounted on dedicated linear stages that run across a large granite sample table (Fig. 1 ▸). This enables each experiment to be brought into the beam separately, such that X-ray transmission powder diffraction data can be collected *via* a large area detector (currently a Pixium RF4343, Thales), which is mounted on a linear drive that runs along the length of the sample table, allowing a predefined sample-to-detector distance to be set. Data collections are made weekly during each synchrotron machine run (∼6–8 weeks) and the wavelength and sample-to-detector distance each week are obtained by refinement of a standard CeO_2_ calibrant that is either built into each sample cell or permanently mounted on the sample stage. During machine shutdown periods (typically ∼4 weeks), although X-ray data cannot be collected, parametric data (*e.g.* cell temperature) and ongoing control and status monitoring are continuously recorded and archived.

To study slow mineral precipitation in cold aqueous environments using the I11 LDE facility, a bespoke transmission-geometry cold-cell that is capable of achieving very slow cooling ramp rates has been designed. The cell can house five separate aqueous samples, of varying ionic composition, allowing the dynamic changes between ice, brine and mineral phases to be investigated simultaneously over the same controlled temperature range [room temperature to −30°C (243 K)]. In this paper, we describe the cell design and report results from a year-long study of the low-temperature precipitation of the mineral phase meridianiite (MgSO_4_·11H_2_O) from an MgSO_4_-rich solution. Meridianiite is believed to be widespread on the present-day surface of Mars and is a diagnostic for the loss of liquid water within a freezing, rather than evaporative, environment (Kargel, 1991 ▸; Hogenboom *et al.*, 1995 ▸).

## 2. Cell design   

### 2.1. Mechanical design   

**Figure 2:**
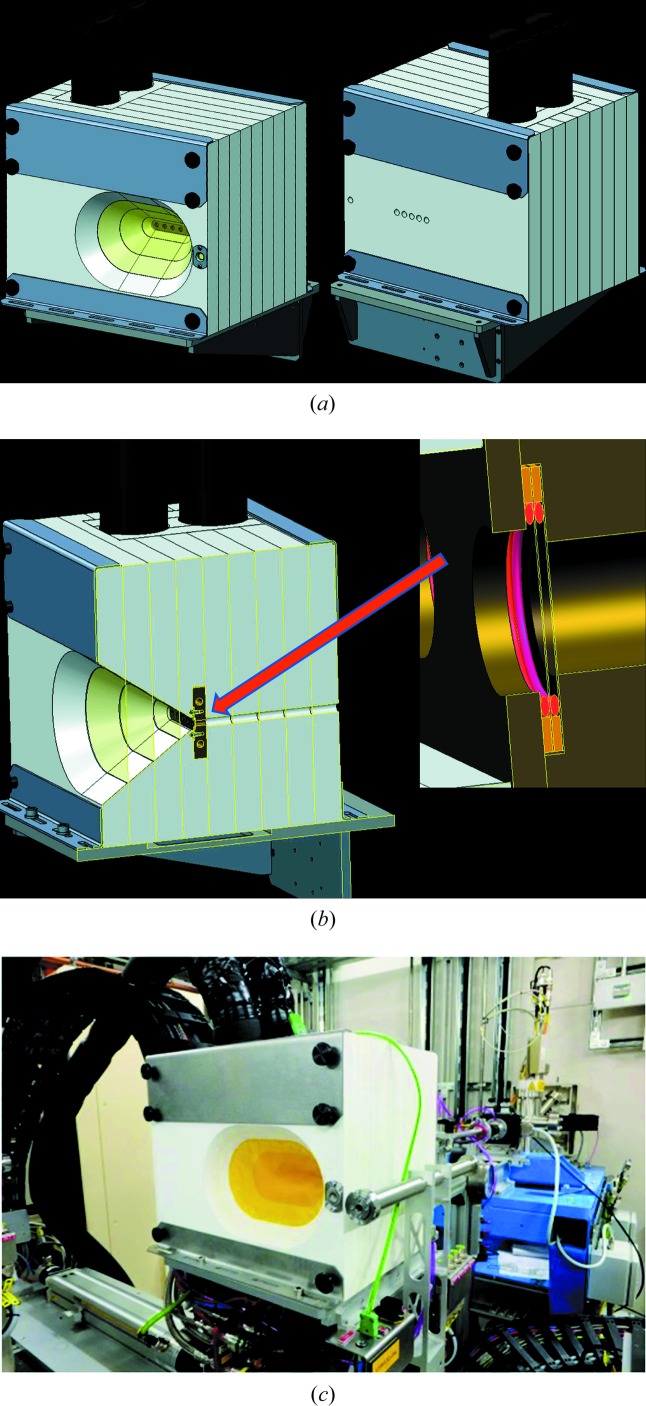
(*a*), (*b*) CAD schematics, showing (*a*) the front and rear (beam exit and entrance) sides of the cold-cell design, and (*b*) the internal construction. (*c*) A photograph of the cell installed on the I11 beamline LDE facility.

Computer-aided design (CAD) renderings and a photograph of the installed cell are shown in Fig. 2 ▸. At the heart of the cell, the sample chamber consists of two bolt-together solid copper body components with two 6 mm diameter circular apertures for beam transmission. A 60:40 glycol antifreeze and demineralized water refrigerant flows in a closed circuit through the blocks *via* an external Lauda ECO RE1050 chiller unit (GOLD control head). The small-volume sample compartment is formed by two 0.05 mm thick diamond windows and two 0.5 mm thick silicone O-rings with an inner diameter of 7 mm, such that the sample fluids do not come into direct contact with the copper body (see inset in Fig. 2 ▸ *b*). The sample block is enclosed within an insulating body made from nine horizontally stacked layers of 25 mm thick Palight PVC foam board, each with a 0.025 mm Kapton window to give an insulating multi-glazing effect while still allowing the transmission of the incident and diffracted beams. The insulating layers are held together by brackets and through-bolts top and bottom. The overall outside dimensions of the cell are 300 mm (width) × 250 mm (height) × 225 mm (length). The cell was mounted on one of the ‘medium’-sized sample stages (see Murray *et al.*, 2017 ▸, for details) on the large sample table in the I11 LDE hutch. Insulated services provide connection to the chiller unit located under the sample table, while a thermocouple embedded in one of the copper blocks connects to patch panel connections built into the sample stage itself. Since it is undesirable to thermally cycle the wavelength calibration standard, this is mounted to one side on the front Palight board with its own dedicated beam path through the upstream boards. There is a fixed 88.46 mm offset along the beam between the sample and standard positions.

The eutectic freezing of concentrated aqueous solutions results in pure-phase water ice, with any salts that were dissolved in the original liquid water being confined to saturated liquid brines, which form inclusions or channels that run through the mass of the frozen ice (Kutschan *et al.*, 2010 ▸). Thus, during the course of the experiment the 7 mm diameter size of the sample compartment means that the cell can be scanned both vertically and horizontally through the beam in order to provide representative average sampling, or to identify the brine-rich regions in which low-temperature mineral precipitation occurs.

### 2.2. Temperature control   

The cell temperature is regulated *via* the Lauda chiller unit which is controlled externally – in open loop – *via* a computer script running on a dedicated EPICS input/output controller (IOC) within the beamline controls system. This provides a programmable smooth decrease in temperature over a 24 h period. Given a starting temperature, ramp rate and the total number of days over which the cell is to be cooled, the script calculates and updates the required chiller setpoint temperature every 5 s. No closed feedback exists between the cell temperature and the chiller temperature (*i.e.* there is no cell temperature dial-up capability) since the intended aim is to provide a selectable linear ramp rate, irrespective of the actual temperature at any one time. The cooling rate of 0.3°C d^−1^ used in the work presented here was selected as it is broadly representative of the seasonal rate of temperature change during Arctic sea ice formation (Cox & Weeks, 1988 ▸) and also allows a reasonable difference in temperature to be achieved within the weekly measurement regime of the I11 LDE facility.

**Figure 3:**
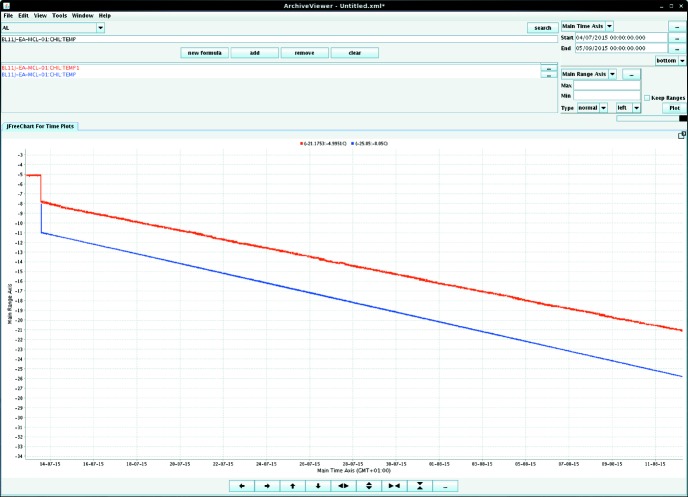
A screenshot showing the cell temperature (red, top trace) and chiller temperature (blue, bottom trace) during a script-controlled cooling ramp covering a 1 month period. The difference between the measured sample temperature and the chiller temperature is initially ∼5°C, widening slowly as the temperature decreases.

Repeated test runs showed a near-perfect linear ramp for both the chiller coolant temperature and the cell temperature (Fig. 3 ▸), with a very gradually increasing offset difference between the two of ∼5°C over the entire operating range. However, this coolant–cell difference and the achieved final base temperature were found to be sensitive to the cooling rate, and a rate of ∼0.5°C d^−1^ or less allowed base temperatures below −29°C (244 K) to be obtained. This matched the original design specification of −30°C (243 K), intended to cover the region of formation of hydohalite (NaCl·2H_2_O), the most abundant mineral formed in terrestrial sea ice below −22°C (251 K) (Butler *et al.*, 2017 ▸). However, faster ramp rates (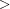 ∼1°C d^−1^) resulted in raising the achievable base temperature by several degrees.

Since the chiller is driven in open loop, in its current form the script applies a specific ramp rate for a set number of days, after which the achieved temperature can be maintained, or the sample thermally cycled by ramping up and down. Furthermore, as it is unlikely that an experiment running in a central facility environment continuously for one or more years will escape experiencing unforeseen power cuts, the script can also be stopped, edited and restarted, during which time the chiller maintains its last received setpoint temperature. Similarly, if, for whatever reason, network communications are lost between the IOC running the script and the chiller (or indeed there is a loss or failure of the IOC itself), the chiller again maintains its last setpoint temperature. The open-loop script approach thus provides a high degree of flexibility of control and ongoing management of the experiment. Backup power to the LDE facility itself similarly ensures continued electrical supply to the chiller in the face of power outages, planned or otherwise.

## 3. Experimental details   

### 3.1. Commissioning experiment: precipitation in the MgSO_4_–H_2_O aqueous system   

Evidence for the presence of magnesium sulfate in Martian soils and rocks first emerged from the Viking landings in 1977 (Clark & Van Hart, 1981 ▸; Clark, 1993 ▸) and was subsequently confirmed by later missions: the Pathfinder landers in 1997 (Rieder *et al.*, 1997 ▸; Wänke *et al.*, 2001 ▸) and the Mars Exploration Rovers, Opportunity and Spirit, in 2004 (Gellert *et al.*, 2004 ▸; Rieder *et al.*, 2004 ▸). *In situ* chemical analyses during these missions showed a strong correlation between sulfur and magnesium, pointing to magnesium sulfate being present in significant quantities. Later, orbital observations showed hydrated sulfate outcrops, several kilometres thick, in the walls of Valles Marineris (Bibring *et al.*, 2006 ▸). Although kieserite (MgSO_4_·H_2_O) was the first phase to be considered likely (Baird *et al.*, 1976 ▸), data from the Mars Express spacecraft (Gendrin *et al.*, 2005 ▸) and the Mars Reconnaissance Orbiter (Roach *et al.*, 2008 ▸) pointed to more highly hydrated sulfates. Indeed, observations show salts (sulfate, chloride and perchlorate) to be widespread across the planet’s surface (*e.g.* Gendrin *et al.*, 2005 ▸; Squyres *et al.*, 2006 ▸; Wang, Haskin *et al.*, 2006 ▸; Hecht *et al.*, 2009 ▸; Massé *et al.*, 2010 ▸; McClennan, 2012 ▸). The presence of MgSO_4_ on Mars is believed to have arisen from the reaction of basaltic material with sulfuric acid of volcanic origin and subsequent evaporation (Tosca *et al.*, 2005 ▸).

Data from the Mars Odyssey Orbiter (Feldman, Prettyman *et al.*, 2004 ▸; Feldman, Mellon *et al.*, 2004 ▸; Feldman *et al.*, 2011 ▸) allowed the global distribution of water-equivalent hydrogen (WEH) down to 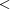
1 m depth to be measured. In two large regions near the Martian equator and in mid-latitude deposits buried in a large region of Arcadia Planitia, WEH concentrations of ∼50 wt% were found, much higher than could be accommodated by open pore-space volumes within the surface regolith. Three possible WEH carriers are thus likely (Feldman, Prettyman *et al.*, 2004 ▸; Feldman, Mellon *et al.*, 2004 ▸; Feldman *et al.*, 2011 ▸): (i) ice, (ii) water adsorbed by mineral grains and (iii) hydrous minerals. At mid to low latitudes ground ice is unstable and, while phyllosilicates and zeolites with 5–21 wt% structural H_2_O/OH are common, their observed abundances cannot account for the observed WEH. However, with up to 62 wt% structural H_2_O/OH, sulfates require less surface material to be present in order to account for the observed WEH. Although rare on Earth and limited to a few – mostly glacial and sea ice – occurrences, the undecahydrate sulfate meridianiite (MgSO_4_·11H_2_O) has the highest hydration state of all the magnesium sulfates and, under present-day conditions, chemical models predict meridianiite to be the most abundant and widespread hydrous sulfate phase on the Martian surface (Toner *et al.*, 2014 ▸). Indeed, meridianiite derives its mineralogical name from the Opportunity rover landing site in Meridiani Planum (a plain located 2° south of the Martian equator), where its presence was deduced *in situ* from rover observations (Peterson & Wang, 2006 ▸; Squyres *et al.*, 2006 ▸). Meridianiite forms at low temperatures as a precipitate from freezing staturated MgSO_4_–H_2_O solutions (Kargel, 1991 ▸; Hogenboom *et al.*, 1995 ▸).

Laboratory studies and thermodynamic modelling show that liquid water could theoretically exist – at least transiently – on the present-day Martian surface (Brass, 1980 ▸; Haberle *et al.*, 2001 ▸; Knauth & Burt, 2002 ▸; Chevrier & Altheide, 2008 ▸; Chevrier, Ulrich & Altheide, 2009 ▸; Chevrier, Hanley & Altheide, 2009 ▸; Möhlmann, 2011 ▸; Gough *et al.*, 2011 ▸), but because the surface conditions are close to the triple point of water, pure liquid water is likely to be unstable (Haberle *et al.*, 2001 ▸). However, the presence of salts should lower both the freezing point and the evaporation rate of water. As a consequence, brines should be more stable and are therefore more likely to be present on the Martian surface (*e.g.* Brass, 1980 ▸; Altheide *et al.*, 2009 ▸; Zorzano *et al.*, 2009 ▸; Möhlmann & Thomsen, 2010 ▸). These could have become systematically enhanced in Ca, Mg or Na, depending on whether they are in contact with the CO_2_-rich Martian atmosphere or isolated in the sub-surface region (Knauth & Burt, 2002 ▸; Burt & Knauth, 2003 ▸). Experiments have shown halotolerant and sulfate-reducing bacteria can survive in highly concentrated sulfate-rich brines (Marnocha *et al.*, 2011 ▸; Crisler *et al.*, 2012 ▸). Under freezing conditions, the precipitation of hydrated mineral phases such as meridianiite from these enriched brines will occur. Consequently, the melting of meridianiite may be the most likely source of transient liquid water on the present-day surface, especially at low latitudes where ice is less common in the regolith (Chou & Seal, 2007 ▸), and may contribute to the formation of the chaotic outflow terrains observed on the Martian surface (Peterson & Wang, 2006 ▸).

Understanding the precipitation of hydrated MgSO_4_·*n*H_2_O phases such as meridianiite thus impinges on understanding both the present-day hydrological cycle on Mars and the formation and distribution of relic hydrated phases associated with the historic loss of Mars’ ancient oceans. Therefore, as a commissioning experiment for the LDE cold-cell, we monitored over a period of one year the behaviour of a slowly cooling MgSO_4_–H_2_O solution formulated to precipitate MgSO_4_·11H_2_O. During this time we observed the precipitation of two hydrated MgSO_4_ phases and a disordered ice/sulfate phase and monitored their long-term dynamic behaviour.

### 3.2. Sample preparation, data collection and reduction   

In what follows we will, for ease of comparison, refer to the various MgSO_4_·*n*H_2_O phases as MS*n*, such that meridianiite, with 11 waters of hydration, will be MS11, epsomite (MgSO_4_·7H_2_O) will be MS7 *etc*. When necessary, the use of mineral names will be restricted to the scientific discussion and their MS*n* given in parentheses.

For the commissioning experiment, an aqueous solution of 8 wt% concentration of MgSO_4_ was made by mixing 5 g of reagent grade MS7 with 24 g of 18 MΩ cm deionized water. With part of the copper block assembly on its back and a diamond window and O-ring in place, the solution was added dropwise into the O-ring centre using a pipette. The second window and O-rings were inserted, followed by the second half of the copper body, and the cell was assembled and mounted on the beamline (Fig. 2 ▸).

Prior to the start of the experiment, the detector was set to a nominal 350 mm from the sample, sufficient to give a reasonably wide 2θ range of 42°, and the centre position of each cell was determined by scanning the cell chamber both horizontally and vertically through the beam. This position was entered into a data-acquisition script such that the chamber returned to the same sampling position each week. Diffraction data were then collected each week (see Murray *et al.*, 2017 ▸, for details) at this optimal in-beam position and, for the present work, at a number of plus-or-minus vertical positions offset from the centre by 1–2 mm. The beam size was 400 × 400 µm, achieved by slitting down the incident 25 keV X-ray beam. Each week, using data from the CeO_2_ reference standard, the wavelength and detector distances were refined by the beamline data-acquisition and -reduction pipeline that incorporates the *DAWN* software suite (Filik *et al.*, 2017 ▸), which was also used to integrate and convert the two-dimensional images collected from each cell position to conventional 2θ *versus* intensity ASCII files.

In this type of aqueous brine system and environment, ice formation can be problematic for the collection of powder diffraction data. In particular, the formation of large ice crystals can result in very intense diffraction spots occupying a significant area of the detector. While it is, in principle, possible to mask these out prior to integration, the volume of data accumulated and the variability in their occurrence from week to week (such that a new mask would need to be drawn for each individual image) meant that this was not a feasible option in the first instance (see *Discussion* [Sec sec5] section for future developments). Simple thresholding produced variable results such as halo artefacts, missed spots and loss of legitimate signals. Instead, all images were integrated and the one-dimensional data plotted together on the same axes. Those data sets collected at different positions showing large, intense and wide non-background features were rejected, while the remaining data were averaged, thus producing a single data file for each week.

## 4. Results   

### 4.1. Long-term behaviour of the MgSO_4_–H_2_O system   

**Figure 4:**
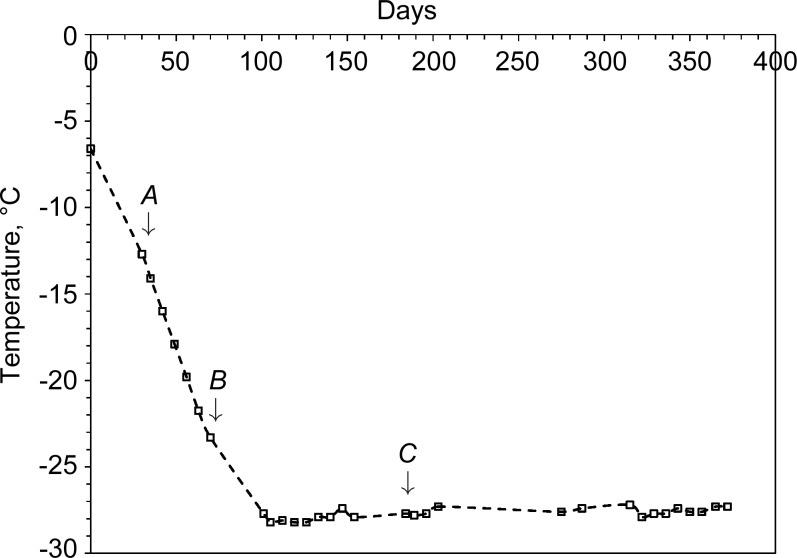
Cell temperatures at various data-collection dates. Gaps are due to synchrotron shutdowns. Arrows *A*, *B* and *C* indicate 30, 70 and 184 days (*T* = −12.7, −23.3 and −27.7°C, respectively; 260.5, 249.9 and 245.5 K, respectively) for comparison with data shown in Figs. 6 and 7.

**Figure 5:**
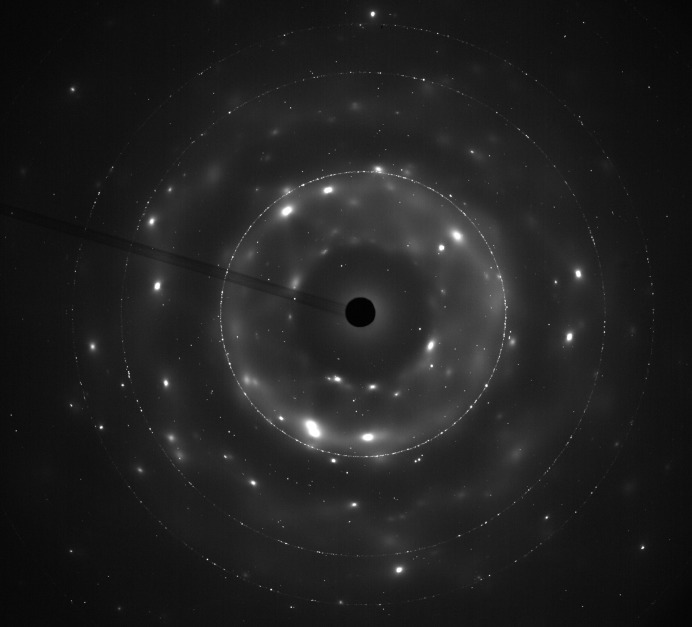
Area-detector image collected at point *B* [70 days, *T* = −23.3°C (249.9 K), λ = 0.49392 Å, detector distance = 335.097 mm] in Fig. 4 ▸, showing the presence of diffuse scattering.

**Figure 6:**
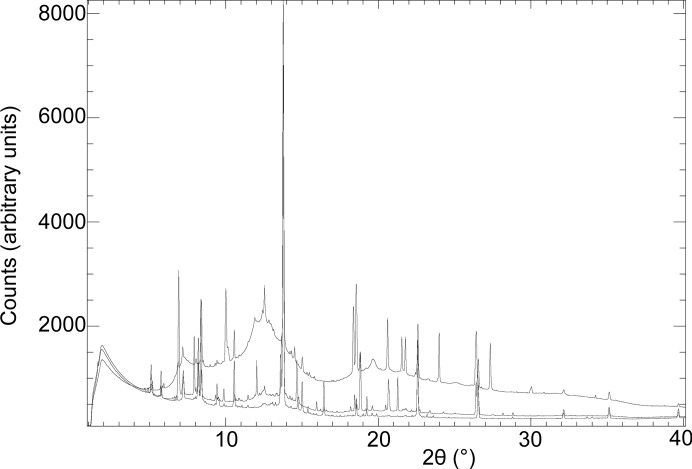
Diffraction patterns of the MgSO_4_–H_2_O system obtained after (bottom to top) 30, 70 and 184 days [*T* = −12.7, −23.3 and −27.7°C, respectively (260.5, 249.9 and 245.5 K, respectively); λ = 0.49383, 0.49392 and 0.49412 Å, respectively; detector distance = 335.153, 335.097 and 335.072 mm, respectively]. Patterns correspond to points *A*, *B* and *C*, respectively, in Fig. 4 ▸ and show the increase in diffuse background scatter.

**Figure 7:**
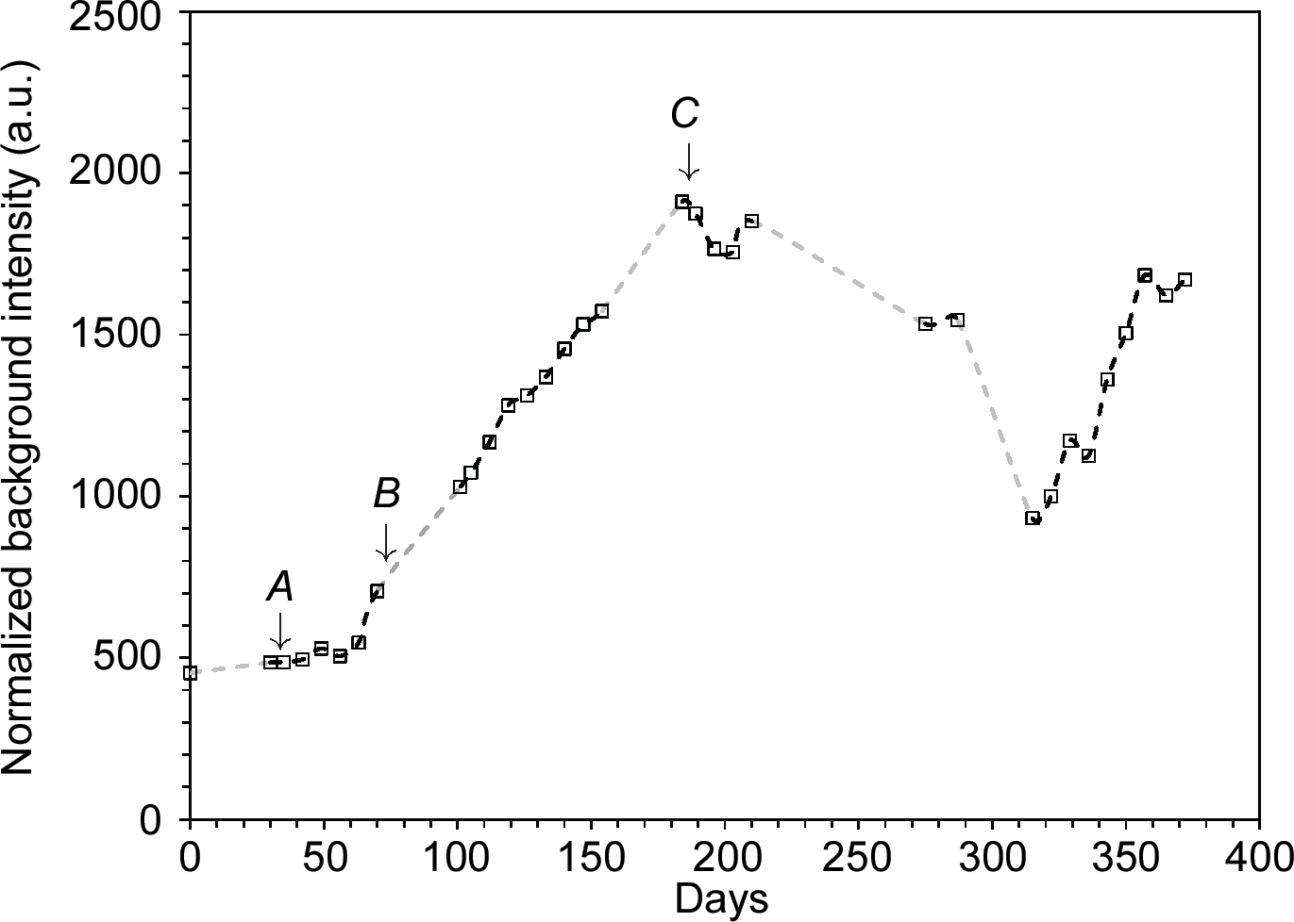
Diffraction background signal at 12.5° 2θ, normalized to the integrated intensity of the CeO_2_ standard, plotted as a function of time. Arrowed points as per Fig. 4 ▸.

In order to start from an ice–brine system, the cold-cell was supercooled to −8°C (265 K) to promote initial freezing, after which the temperature was ramped at −0.3°C per day under computer control. Taking this as day 0, Fig. 4 ▸ plots the measured cell temperatures on the days X-ray data were collected, while Fig. 5 ▸ shows a typical image collected from the area detector. In this figure can be seen (i) relatively good powder rings due to the cell’s diamond windows, (ii) very spotty rings due to a combination of crystalline ice and sulfate precipitate, and (iii) diffuse scatter coming from an amorphous component. In Fig. 4 ▸ three key points, *A*, *B* and *C*, are indicated, corresponding to 30, 70 and 184 days. The corresponding one-dimensional integrated and averaged diffraction patterns for these are shown in Fig. 6 ▸. In this figure, the large Bragg peak just below 14° 2θ is diamond (as are smaller features at ∼22.5, 26.5, 32.2 and 35.2° 2θ), with other Bragg peaks being due to hexagonal ice and sulfate precipitate. There is also a very clear difference in the background signal. Since the pattern at *C* was collected at a much lower temperature than the pattern at *A*, and after some considerable time, this suggests a time/temperature evolution. Fig. 7 ▸ plots the intensity of the background signal at 12.5° 2θ as a function of time. This angle was selected because it represents a high point in the background and does not coincide with any Bragg features in the data. To account for the variation in beam intensity from week to week due to variations in the synchrotron beam fill and small errors in the repeatability and alignment of the beamline optical components, the measured background intensity was normalized to the total scattered intensity of the CeO_2_ standard attached to the cell. This reveals an interesting behaviour. Firstly, the background only starts to increase once point *B* in Fig. 4 ▸ is reached after ∼70 days. Secondly, the background increases to a peak value at point *C* and then decreases, reaching a minimum somewhere between 290 and 320 days (the large gaps between points being due to machine shutdowns), after which it increases in intensity again. Fig. 4 ▸ identifies point *B* as the point when the cell temperature dropped below −22°C (∼251 K). Below this temperature, the number of Bragg peaks in the integrated patterns also increased and could be fitted by a mixture of hexagonal ice, MS7 and MS11 (see §4.3). The rise in the strength of the background is caused by the precipitation of sulfate phases from the brine. The associated removal of Mg^2+^ and SO_4_^2−^ promotes instantaneous ice formation to re-establish ice–brine equilibrium at these low temperatures, which concentrates the brine and results in further MgSO_4_·*n*H_2_O precipitation. This cycle propagates in tandem until ice–brine–MgSO_4_ equilibrium is attained. Because the temperature is so low the precipitation of ice occurs rapidly, resulting in a disordered ice phase and an increase in the diffuse scatter.

### 4.2. Sample temperature   

Fortes *et al.* (2008 ▸) analysed the thermal expansion behaviour of MS11 from 250 to 4 K in terms of the Einstein oscillator model for the specific heat, and we use this here to show that the temperature achieved by the sample is accurately represented by the temperature recorded by the thermocouple embedded in the cell body. The Einstein model assumes that all atoms in the solid vibrate at the same characteristic angular frequency ω_E_, expressed in terms of an Einstein temperature, θ_E_ = 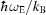. Thus, the values of each of the MS11 lattice parameters *X* at a given temperature *T* can be expressed relative to their values at absolute zero *X*_0_ by 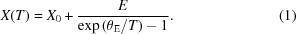
Here, *E* is a constant given by 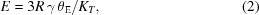
where γ is the Grüneisen ratio relating the volume thermal expansion coefficient α_*V*_, the specific heat capacity *C*_*V*_, the molar volume *V*, the gas constant *R* and the isothermal bulk modulus *K*_*T*_. Assuming γ and *K_T_* are independent of temperature, the thermal expansion behaviour is then a function of the internal energy of the crystal *via* its heat capacity. Although not as rigorous as the Debye formalism, the Einstein model has [as noted by Fortes *et al.* (2008 ▸)] successfully been used to model various related inorganic solids such as CaSO_4_·2D_2_O (Knight *et al.*, 1999 ▸) and is mathematically simple. Fortes *et al.* (2008 ▸) parameterized *E* by a polynomial function, 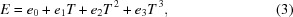
obtaining values for the *e_n_* coefficients from fits to their measured data. Since the temperature range of the cold-cell overlaps the temperature range covered by the Fortes *et al.* (2008 ▸) neutron study, we used their values of *e_n_* and θ_E_ to calculate expected values for the meridianiite lattice parameters near the base temperature recorded by the cold-cell thermocouple [−23 to −28°C (250 to 245 K)] and compared them (Table 2 ▸) with values obtained from fits (see next section for details) to the cold-cell diffraction data collected at these temperatures. Given the limitations of the Einstein model, the agreement between the calculated and fitted parameters is within ∼1% and shows that the temperatures being achieved by the sample within the cell are well represented by the thermocouple measurement, confirming the cell design and its appropriateness for this type of medium- to low-temperature study.

### 4.3. Data analysis: ice formation and sulfate precipitation   

**Figure 8:**
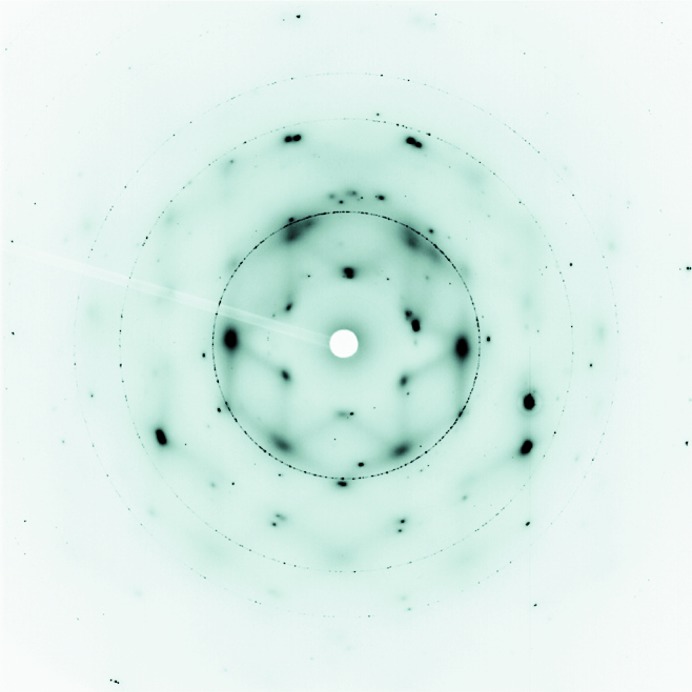
Area-detector image collected at point *C* [184 days, *T* = −27.7°C (245.5 K), λ = 0.49412 Å, detector distance = 335.072 mm] in Fig. 4 ▸, shown in negative to emphasize the symmetry of the diffuse scatter, which exhibits very similar symmetry to the diffuse scatter observed in hexagonal ice with static disorder (Wehinger *et al.*, 2014 ▸).

A major problem in the MgSO_4_–H_2_O system is the nature of the precipitate. Many of the possible MgSO_4_·*n*H_2_O phases (*n* = 1, 2, 3, 4, 5, 6, 7, 11) share the same unit cell structures (see Table 3 ▸) and during profile refinement, owing to the large numbers of closely spaced reflections, can almost all fit the same medium-resolution data out to high angle. Thus, to distinguish between precipitated phases, we restricted our analysis to low angles where the differences in cell dimensions offer the prospect of distinct diffraction-peak positions (see also the discussion in §5.2 for improvements to data collection). Conveniently, the cubic unit cell of the diamond windows yields a strong and narrow Bragg peak at ∼14° 2θ and provides both a useful cut-off and an additional intensity calibrant (see below). We also used this feature to refine the 2θ zero point, allowing the diamond lattice parameter to float to account for the change in temperature. The similarity of the pattern of diffuse background scattering recorded in the area-detector images to patterns of hexagonal ice with static disorder [*e.g.* compare Fig. 8 ▸ in the present work with Fig. 1 in the paper by Wehinger *et al.* (2014 ▸)] meant that our first step in obtaining Pawley fits to the low-angle diffraction data was to include two hexagonal ice phases, one to account for the narrow Bragg features of crystalline ice and the other to account for the broad diffuse contributions. Following this, profiles for MS7 and MS11 were first refined separately and then together to obtain the relative contributions of each phase. However, in doing this we found that the fits could be improved by including an amorphous sulfate phase (sometimes two) to better model the background, suggesting that the rise in diffuse scatter may also contain contributions from amorphous or nanosized sulfate. Although it is unrealistic to suggest that an amorphous sulfate component can be identified in this way as, in the fit, it is really only accounting for deficiencies in the background function, we found that certain phases gave better improvements than others. For example, amorphous MS2, MS4 and MS5 tended to provide improvements for the earlier data, while MS4, MS5 and MS6 gave improvements for data collected later in the run. Including amorphous MS1 and MS3 rarely gave improvements and adding in additional MS7 or MS11 phases never improved the fit. However, note that the formation of amorphous MS2 and MS3 from MS7 under desiccating conditions at −8°C (265 K) has previously been observed (Wang, Freeman *et al.*, 2006 ▸): although these are amorphous by X-ray diffraction, they were readily identifiable by the position of the strong Raman feature near 1000 cm^−1^.

**Figure 9:**
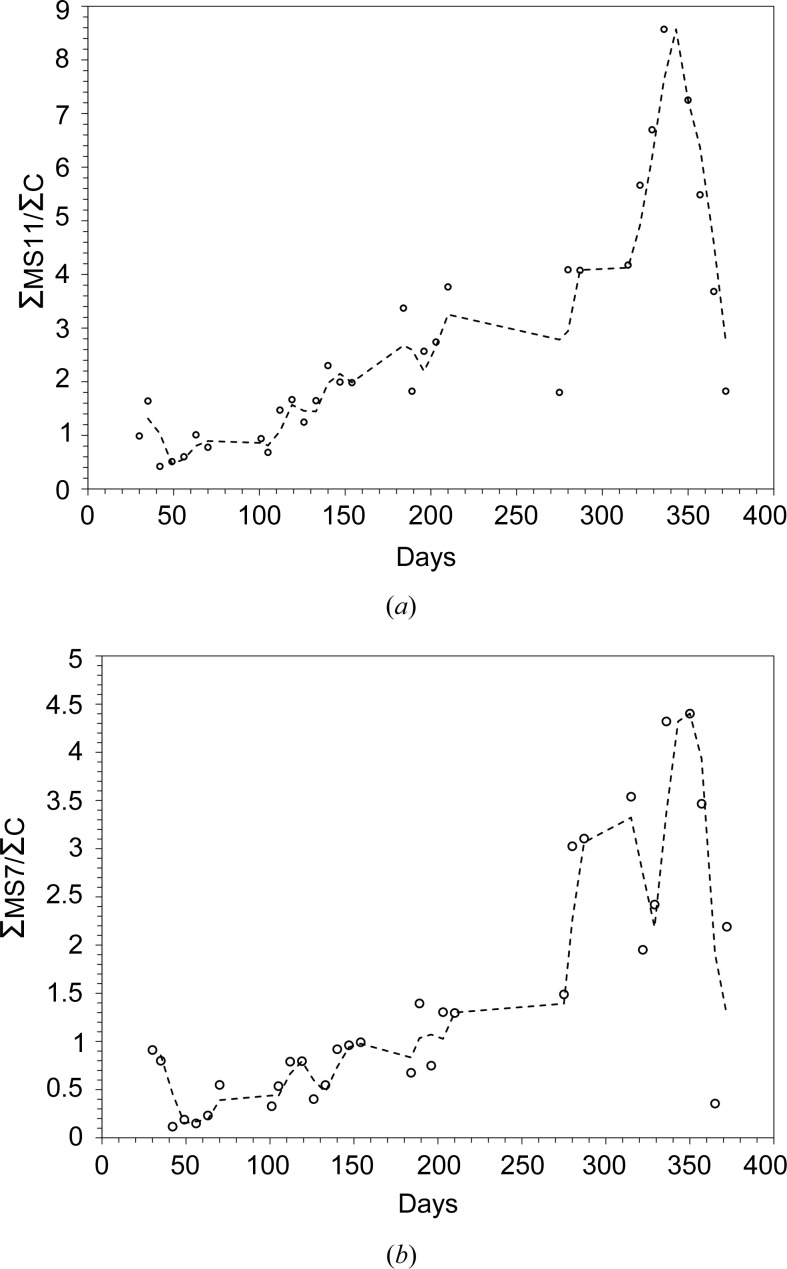
Ratios of the area of the diffraction fit attributed to (*a*) MS11 and (*b*) MS7 to the area of the first diamond peak from the cell window. Dotted lines are two-point moving averages to highlight the trend.

To gain a picture of how the precipitate system evolved with time, the area under the fit for each component was normalized to the area of the 14° 2θ diamond feature, and these are shown in Fig. 9 ▸. Interestingly, a peak in both MS11 and MS7 is observed at ∼340 days, which immediately follows the minimum seen in the background signal (Fig. 7 ▸).

Over the course of the experiment we have thus observed the following sequence: formation of a first ice phase as the system initially cools, followed at ∼70 days, once the cell temperature has dropped below ∼23°C (250 K), by the simultaneous precipitation of MS7 and MS11 and the formation of a disordered phase. The disordered phase continues forming, while the MS7 and MS11 levels increase little, if at all. However, after 100 days the levels of MS7 and MS11 start to increase, until at ∼280 days MS7 increases more steeply, followed at ∼320 days by MS11. Meanwhile, at ∼200 days the disordered phase starts to decrease, reaching a minimum somewhere around 300 days, after which it starts to increase again. At the end of the first MS7/11 growth period, the ratio of MS11:MS7 is approximately 4:1, while after the second it is approximately 2:1, suggestive of some ongoing processing relationship between the sulfate, ice and/or the disordered phase.

## 5. Discussion   

### 5.1. Sulfate precipitation sequence   

**Figure 10:**
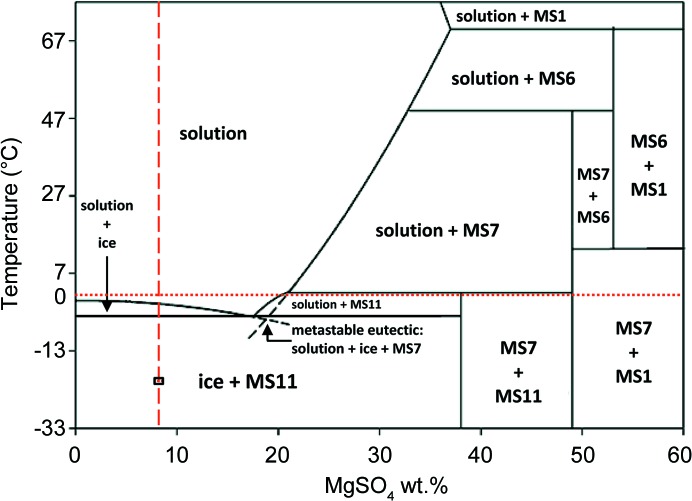
The phase diagram for the H_2_O–MgSO_4_ system [after Hogenboom *et al.* (1995 ▸), Peterson & Wang (2006 ▸) and Fortes *et al.* (2008 ▸)]. The red vertical dashed line represents the 8 wt% starting composition used in this work, and the black square superimposed on this line represents the achieved base temperature of the cell.

The 8 wt% MgSO_4_ concentration and base temperature [−28.2°C (245 K)] are marked on the MgSO_4_–H_2_O phase diagram shown in Fig. 10 ▸. However, the MgSO_4_–H_2_O phase diagram derives from those presented by Kargel (1991 ▸) and Hogenboom *et al.* (1995 ▸), which are based on much earlier studies [citations in Kargel (1991 ▸) and Hogenbloom *et al.* (1995 ▸)] combined with results obtained by Hogenboom *et al.* (1995 ▸) [note that, until Peterson & Wang (2006 ▸) solved the structure by single-crystal X-ray diffraction and confirmed that the asymmetric unit contains only 11 water molecules, MS11 was misidentified as MS12 in earlier work].

Tracing down the 8 wt% line superimposed on the MgSO_4_–H_2_O phase diagram suggests the sequence of 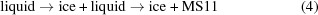
should be observed. At 0.1 MPa (atmospheric pressure) the initial ice phase should be hexagonal ice, I*h*, which we observe. However, the MgSO_4_–H_2_O equilibrium phase diagram does not indicate the formation of a disordered phase or MS7. Our observed sequence is 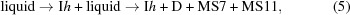
where D represents the phase responsible for the diffuse scattering. Although Hogenboom *et al.* (1995 ▸) did not explore the behaviour of an 8 wt% solution at atmospheric pressure, they did observe the precipitation of metastable MS7 in the MS11 stability field for higher concentrations (*e.g.* 22 wt% solution) and at high pressures. They also noted that MS11 forms more slowly than MS7 [also noted by Kargel (1991 ▸) at atmospheric pressure] and may be related to the difficulty in achieving equilibrium crystal nucleation during cooling of the saturated MgSO_4_ solution. This phenomenon is common among binary and multicomponent systems and especially so in MgSO_4_ solutions (Kargel, 1991 ▸), occurring when the cooling rate is too fast and the system supercools. This suggests that, even at a cooling rate of 0.3°C d^−1^, the MgSO_4_–H_2_O system within the cold-cell is still out of equilibrium. However, we do note that, in order to promote the first ice freeze and to form liquid brines within the ice, the cell was fast ramped to −8°C (265 K) over the course of ∼1 h. The first ice freezing will increase the wt% concentration of MgSO_4_, moving the red dashed line in Fig. 10 ▸ to the right. When the temperature drops to −23°C (250 K) and MS7 and MS11 start precipitating out, the formation of the disordered phase (which we assume is a mixture of disordered ice and amorphous MS) concentrates the brine still further. Thus, even though MS11 is thermodynamically favoured and initially forms in greater quantities (*i.e.* 4:1), the faster growth rate for MS7 means that by the end of the observed growth period the ratio of MS11:MS7 has reduced to approximately 2:1. The fact that we observe two different growth phases for both MS7 and MS11 points to a complex interplay between the precipitated phases and the trapped brine.

Towards the end of the experiment, at ∼340 days, MS11 and M7 reach a peak and start to decline, while the background signal, which had previously reduced in intensity, has been increasing since ∼310 days. Although we have no definitive explanation at this point, it may be possible that the sulfate phases reach a point where they are unstable with respect to the remaining brine and begin re-dissolving, releasing their waters of hydration which, owing to the low temperature, rapidly freeze as part of the disordered phase. Whether this process results in nanoscale or amorphous MS4/MS5/MS6 components (as suggested in §4.3), derived from the excess MS11/7, being incorporated into the disordered phase remains to be seen. If this explanation holds true, then we might predict that this growth-and-decline behaviour is part of a longer-term behaviour, akin to damped oscillations, as the system eventually moves towards equilibrium. Operational constraints unfortunately meant that we were unable to run the experiment beyond a year.

### 5.2. Future upgrades and improvements   

As discussed above, the similarity in structure of many of the precipitating salt systems means that resolving closely located or overlapping diffraction features is important. In the I11 LDE setup there are several limiting factors that it may be possible to improve or optimize.

#### 5.2.1. Resolution   

Small beam sizes are currently achieved by slitting down the beam to improve resolution, and this is obviously at the expense of incident intensity, requiring longer collection times to compensate. To overcome this, an F-switch ‘random access’ compound refractive lens manipulator (Duller *et al.*, 2016 ▸) is currently being commissioned. Based on the transfocator concept (Vaughan *et al.*, 2011 ▸), the F-switch houses 120 selectable compound refractive lenses which can be chosen to provide selectable spot sizes at a given position, typically from 100 µm down to 5 µm diameter, while still providing a gain in intensity and a reduction in data-collection times. This in turn will provide for either increasing the number of spatially resolved points across the sample at which data are collected, or the direct sampling of individual brine inclusions and channels. A further advantage of introducing a focusing element will be to increase the divergence of the incident beam over that of the undulator beam, which should improve powder averaging around the diffracted Debye–Scherrer rings.

**Figure 11:**
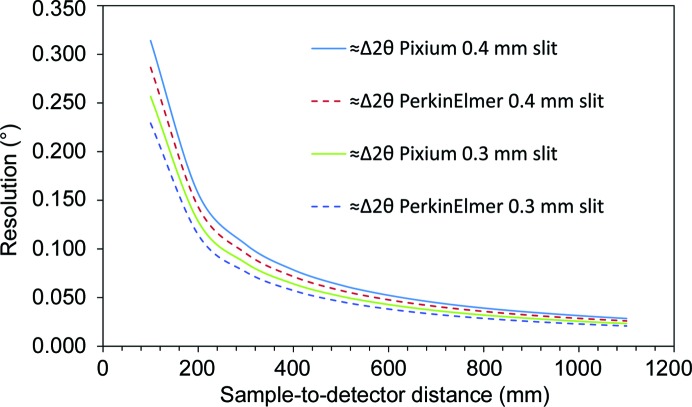
Predicted detector resolution as a function of distance from the sample for two beam sizes defined by square slits of dimension 400 and 300 µm towards the centre of two area detectors, a Thales Pixium 4343 with a pixel size of 150 µm, and a PerkinElmer 4343 with a pixel size of 100 µm.

**Figure 12:**
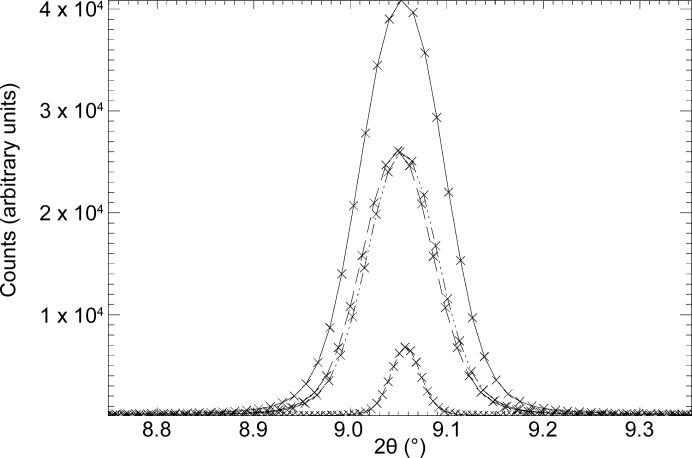
The CeO_2_ 111 peak measured (λ = 0.49319 Å) for different nominal detector distances and beam-size combinations. Strongest trace (solid line): detector distance 350 mm, beam size 400 × 400 µm. Middle two traces (dashed and dashed–double-dotted): detector distance 350 mm, beam size 300 × 300 µm, and detector distance 800 mm, beam size 300 × 300 µm, respectively. Weakest trace (dashed–dotted): detector distance 800 mm, beam size 300 × 300 µm.

Neglecting the sample thickness contribution, for a regular shaped beam of dimension *d*, in transmission from a sample to a detector with pixel size *p* at a distance *D* away, the 2θ resolution (towards the centre of a flat detector) is approximately given by 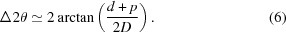
Fig. 11 ▸ shows Δ2θ as a function of detector distance, calculated for the Pixium 4343 area detector which is currently deployed on the I11 LDE and which has a pixel size of 148 µm. For a beam size of 400 × 400 µm, distances of 500 and 950 mm should give Δ2θ of ∼0.063 and ∼0.033°, respectively, while a beam size of 300 × 300 µm should yield ∼0.051 and ∼0.027°, respectively. Also shown in Fig. 11 ▸ is Δ2θ calculated for a PerkinElmer 4343 XRPAD area detector, which has a pixel size of 100 µm. Not available when the LDE facility was constructed, a PerkinElmer detector is currently in commissioning and, at the long detector distance and smaller beam size, should in principle provide further improvement in resolution. Shown in Fig. 12 ▸ are the (Pixium) measured 111 Bragg peaks of the cold-cell’s CeO_2_ reference standard for the same detector distances and beam sizes, obtained by integrating around the ring. At 500 mm from the sample, the FWHMs for the 400 × 400 µm and 300 × 300 µm beam sizes are 0.1 and 0.08, respectively, and are clearly quite large, while at 950 mm the FWHMs are 0.086 and 0.036, respectively, with the latter lying closer to the calculated Δ2θ. The discrepancy at shorter detector distances probably originates from the use of mechanical slits to define the beam, the sample thickness, the increased path length to higher detector angles at shorter distances and the associated increase in the spread of the scattered beam as the scattering angle increases. Fig. 12 ▸ also highlights the decrease in the diffracted signal strength incurred by slitting down the beam to increase resolution.

A detector distance of 950 mm means that the powder ring from the cold-cell’s diamond window is just contained within the active area of the detector, allowing maximum separation of the lower-angle precipitate peaks. However, there is a practical limitation to the maximum usable detector distance, imposed by the need to calibrate the wavelength and the detector distance simultaneously. As the detector distance increases, the number of CeO_2_ rings captured by the detector decreases, such that the uncertainty in the wavelength and 2θ zero point also increase. For 25 keV X-rays, at this distance seven CeO_2_ rings are captured, aided by the fact that the CeO_2_ standard is offset closer to the detector than the sample by 88.46 mm. Increasing this offset could allow for more rings to be captured, but to be useful the standard mount would need to extend outside the sample-stage footprint and thus risk fouling adjacent LDE experiments.

#### 5.2.2. Data reduction and pipelining   

The current data-collection and -reduction pipeline for each weekly beamtime collection consists of the following steps: (i) using the area detector to collect *N* two-dimensional patterns from different positions of the sample; (ii) integrating the two-dimensional detector images to *N* one-dimensional data files; (iii) rejecting those one-dimensional patterns that contain significant contributions from large ice spots; (iv) summing or averaging the remaining one-dimensional files and normalizing to the integrated intensity of the CeO_2_ reference standard. Steps (ii) to (iv) are currently done entirely manually and are dependent on the operator’s sense of what constitutes a ‘bad’ pattern. They are wasteful of data in that a single large spot in a detector image means that the useful information contained in the rest of the image is being discarded. To improve on this, the process ideally needs the individual area-detector images to have a mask applied prior to integration. In normal circumstances, an operator would define the mask position interactively using the reduction software, and this would be applied to all subsequent images. However, in the case of the cold-cell data, not only are the large spots in different places on the image from position to position in the sample, but they also vary from week to week owing to the dynamic nature of the ice–brine system. Automatic detection and masking would solve this problem, but would need to be sufficiently robust that (i) they do not inadvertently remove good data and (ii) they do not introduce artefacts into the data.

### 5.3. Current deployment and future experiments   

Following on from these commissioning experiments, a number of low-temperature aqueous precipitation experiments are in progress, with future experiments planned. Although the outcome of these will be reported in separate publications (*e.g.* Butler *et al.*, 2018 ▸) we highlight some of them here.

#### 5.3.1. Sea ice in Earth’s polar oceans   

As mentioned in the *Introduction* [Sec sec1], the formation of minerals in sea ice has an important role to play from both an ecological and a climatological perspective. However, *in situ* measurement of the composition, distribution and dynamics (solubility, precipitation and dissolution) of authigenic mineral assemblages in freezing ocean waters have only recently begun. This, and the dependence on thermodynamic modelling, which is reliant on interpolation and extrapolation, clearly limit our ability to understand the evolutionary sequence of mineral dynamics in cold environments, where the kinetics may mean that thermo­dynamic equilibrium is not attained or maintained before environmental conditions change. Such environments in Earth’s polar oceans are the least understood in terms of their input to climate change. Compositional changes take place within thickening sea ice, where the ice temperature varies both with depth and with season. The complexity of the slowly evolving sea ice, and changes in brine composition and consequent mineral formation, have not yet been measured using conventional laboratory techniques. The 0.3°C d^−1^ cooling rate used in the commissioning work reported here also represents a good proxy for the change in temperature observed in a natural setting at the ice–air interface in the Arctic ocean during a seasonal cycle of sea ice formation.

Seawater has a typical dissolved salt content (salinity) of ∼35 g kg^−1^ and currently three conservative seawater brines are under investigation using the cold-cell, with salinities of 100, 75 and 35 g kg^−1^. Of particular interest will be the verification of the time minerals such as hydrohalite take to reach equilibrium under conditions that are equivalent to those found *in situ*, within the brine pores and channels of sea ice. A more complete description of the mineralogical evolution of sea ice that these experiments could provide would allow for a quantitative assessment of the contribution of sea ice minerals to the processes driving global warming and cooling cycles, both historically and at the present day.

#### 5.3.2. Ocean worlds in the solar system   

Among the ice and ocean worlds of the solar system, Europa is thought to be unique because observations and models suggest its ocean is in direct contact with its rocky interior. Therefore, the potential chemical composition of the Europan ocean could be constrained through possible weathering/leaching reactions and the assumed composition of its carbonaceous chondrite core. From observational data, the salinity of Europa’s sub-surface ocean is believed to be 3–15 g kg^−1^ (Hand & Chyba, 2007 ▸) and a model Na–Mg–Ca–Cl–SO_4_ solution was prepared gravimetrically with the following ionic composition in 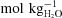: Na = 1.630, Mg = 2.929, Ca = 0.0064, SO_4_ = 3.5964 and Cl = 0.308. The solution was cooled at the same rate as the seawater and brine samples and the expected phases at various temperatures include MS7, CaSO_4_·2H_2_O (gypsum), Na_2_SO_4_·10H_2_O (mirabilite), MS11, water ice and NaCl·2H_2_O (hydrohalite). However, as we have already shown with the MS11 results obtained thus far, precipitate behaviour may not be fully described by the current thermodynamic model.

Other possible future targets include Titan which, apart from Earth, is the only solar system body with a thick N_2_ atmosphere and the only moon to retain a substantial atmosphere. This makes Titan home to a complex atmospheric chemistry and the only other solar system body with stable liquid currently on its surface. The combination of oxygen-bearing molecules and favourable conditions for organic haze formation means that atmospheric processes could yield molecules of prebiotic interest. Similarly, a combination of organics and liquid water in a sub-surface ocean and methane and ethane in surface lakes and oceans makes Titan a prime target for furthering our understanding of prebiotic chemistry, habitability and the potential for life to exist beyond Earth (Horst, 2017 ▸). The presence of a saline sub-surface ocean was confirmed by the Huygens probe which descended to the surface (Béghin *et al.*, 2012 ▸) and by radio Doppler tracking (Iess *et al.*, 2012 ▸), while analysis of Titan’s gravity and topography constrain the sub-surface ocean density to be slightly higher than that of pure water, suggesting the presence of salts (Mitri *et al.*, 2014 ▸), with ammonium sulfate [(NH_4_)_2_SO_4_] being considered a likely constituent (Fortes *et al.*, 2007 ▸; Grindrod *et al.*, 2008 ▸). Such an ocean would have a freezing temperature of approximately −23°C (250 K) (Grindrod *et al.*, 2008 ▸), which is well within the cold-cell’s specification.

## 6. Conclusions   

We have presented the design of a cold-cell for long-duration studies of mineral precipitation from cold aqueous environments and demonstrated its performance. Along with this, we have reported a commissioning experiment using the MgSO_4_–H_2_O system, showing the cell’s capability for uncovering new scientific phenomena. Specifically, we have observed that, under slow 0.3°C d^−1^ cooling, as well as the predicted hexagonal water ice and MgSO_4_·11H_2_O phases, metastable MgSO_4_·7H_2_O and a disordered ice-rich phase are also co-formed. Following this system for one year, using the beamline I11 Long-Duration Experiments facility at the Diamond Light Source (UK), we observed a complex low-temperature dynamic behaviour, relating to the differential growth rates of the two sulfate phases, and the time-dependent growth and variation of the disordered phase caused by changes in ionic concentration brought about by precipitation of the sulfates. We have also discussed how data quality for future experiments could be improved, and outlined some of the current and planned experiments for this facility.

Cold-cell experiments will produce the first *in situ* observations of mineral formation during the evolution of brine chemistry in aquatic environments that are characteristic of oceans on Earth and other planetary objects, and are undergoing freezing over environmentally relevant timescales. Such novel results will contribute to our understanding of the global processes that occur on, and shape, such bodies, and the results will determine how closely solution chemistry is linked to thermodynamic equilibrium and to mineral solubilities currently predicted by the extrapolation of relevant data down to sub-zero temperatures.

## Figures and Tables

**Table 1:** Ocean worlds of the solar system, grouped according to current evidence [drawn from the literature, though see reviews by Hussmann *et al.* (2006 ▸), Nimmo & Pappalardo (2016 ▸), Lunnie (2017 ▸) and Mann (2017 ▸)] The first group are where extensive multi-channel observations, remote sensing by landers and flyby missions unequivocally point to liquid oceans. The second category are where calculations and models using current observational data support the likelihood of there being an ocean, while the third category are objects which, based on known observational properties, could support a (mostly sub-surface) ocean if certain conditions were met, *e.g.* an ocean of sufficient salinity to act as antifreeze, or where internal heating occurs. This third category also includes the larger trans-Neptunian objects (TNOs) where surface ices are observed, a few examples of which are listed.

Supported by observation	
Earth	Circulating surface oceans, polar ice caps
Mars	Polar ice, evidence of global ancient surface ocean
Ceres	Evidence of ancient, possibly surviving, sub-surface ocean
Europa	Ice crust, evidence for sub-surface ocean
Ganymede	Ice crust, may have stacked oceans separated by ice layers
Callisto	Evidence shows global sub-surface fluid, possibly water
Enceladus	Ice crust, evidence (plumes) of saline sub-surface ocean
Dione	Ice crust, evidence for global sub-surface ocean
Titan	Ice crust, evidence for saline global ocean
Pluto	Plausible interpretation of limited observational data

Predicted by theory and modelling	
Triton	Water is lesser constituent of surface ices, ocean predicted
Mimas	Sub-surface ocean proposed to explain orbital wobble
Charon	Possible evidence supporting frozen sub-surface ocean
Oberon	Ice on surface, sub-surface ocean considered possible
Orcus	Evidence of ancient sub-surface ocean, may have survived
Titania	Possible global sub-surface ocean

Plausible under certain conditions	
Tethys	Pure water ice surface, internal structure unknown
Rhea	Sub-surface ocean plausible from known properties
TNOs	
2007 OR_10_	Bulk density, surface colour and spectroscopic evidence consistent with liquid surface water activity, possibly due to tidal heating caused by orbital interactions with moons; models suggest that heating by radioactive decay could support sub-surface oceans in many larger TNOs
Haumea	
Quaoar	
Makemake	
Eris	
Sedna	

**Table 2:** Parametric MgSO_4_·11H_2_O lattice parameters at absolute zero *X*_0_ and at the Einstein temperature θ_E_, and polynomial coefficients *e_n_* for the constant *E* given by equation (2), taken from Fortes *et al.* (2008 ▸); lattice parameters refined from data collected at −23.3 and −28.2°C (249.9 and 245 K) are compared with calculated values at the same temperature using the parametric values for the lattice parameters at absolute zero

	*a* (Å)	*b* (Å)	*c* (Å)	α (°)	β (°)	γ (°)
Parametric						
*X*_0_	6.72740 (5)	6.78187 (5)	17.31589 (14)	88.2126 (9)	89.4499 (9)	62.6109 (8)
θ_E_	340 (3)	179 (11)	80 (6)	79 (170)	195 (109)	185 (23)
*e*_0_	6.9 (1) × 10^−2^	9.6 (19) × 10^−3^	−3.2 (5) × 10^−2^	1.0 (7) × 10^−2^	1 (1) × 10^−1^	2.1 (6) × 10^−1^
*e*_1_	0	9.8 (4) × 10^−5^	2.2 (5) × 10^−4^	−5 (9) × 10^−4^	−1.7 (18) × 10^−3^	−9.1 (39) × 10^−4^
*e*_2_	0	0	−9.4 (24) × 10^−7^	3 (6) × 10^−6^	1.5 (13) × 10^−5^	1.8 (8) × 10^−6^
*e*_3_	0	0	1.6 (4) × 10^−9^	−6 (15) × 10^−9^	−3.8 (31) × 10^−8^	0

Cell at −23.3°C (249.9 K)						
Calculated	6.75	6.81	17.28	88.1	89.4	62.6
Refined	6.72 (2)	6.77 (2)	17.29 (3)	87.8 (1)	89.1 (2)	62.2 (2)
% difference	0.44	0.59	0.06	0.34	0.34	0.64

Cell at −28.2°C (245.0 K)						
Calculated	6.75	6.81	17.29	88.2	89.5	62.7
Refined	6.78 (2)	6.88 (2)	17.36 (4)	86.9 (2)	89.8 (2)	62.8 (3)
% difference	0.44	1.03	0.40	1.47	0.34	0.15

**Table 3:** Lattice parameters of the various MgSO_4_·*n*H_2_O phases used as starting values for fits to cold-cell data Values are taken from International Center for Diffraction Data PDF 4^+^ database.

Phase	Space group	*a* (Å)	*b* (Å)	*c* (Å)	α (°)	β (°)	γ (°)
MS1	*A*2/*a*	7.511	7.611	6.921	90.00	116.17	90.00
MS2	*P*2_1_2_1_2_1_	8.8934	12.440	8.488	90.00	90.00	90.00
MS3	*Pbca*	10.930	12.420	8.200	90.00	90.00	90.00
MS4	*P*2_1_/*n*	7.902	13.594	5.920	90.00	90.89	90.00
MS5	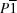	6.335	10.550	6.075	99.17	109.88	75.00
MS6	*A*2/*a*	24.442	7.216	10.119	90.00	98.28	90.00
MS7	*P*2_1_2_1_2_1_	11.910	12.020	6.870	90.00	90.00	90.88
MS11	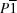	6.732	6.792	17.293	88.22	89.49	62.66
